# Mitotic Spindle Orients Perpendicular to the Forces Imposed by Dynamic Shear

**DOI:** 10.1371/journal.pone.0028965

**Published:** 2011-12-29

**Authors:** Pablo Fernandez, Matthias Maier, Martina Lindauer, Christian Kuffer, Zuzana Storchova, Andreas R. Bausch

**Affiliations:** 1 E27 Zellbiophysik, Technische Universität München, Garching bei München, Germany; 2 Max-Planck-Institut für Biochemie, München, Germany; UT Southwestern Medical Center, United States of America

## Abstract

Orientation of the division axis can determine cell fate in the presence of morphogenetic gradients. Understanding how mitotic cells integrate directional cues is therefore an important question in embryogenesis. Here, we investigate the effect of dynamic shear forces on confined mitotic cells. We found that human epithelial cells (hTERT-RPE1) as well as MC3T3 osteoblasts align their mitotic spindle perpendicular to the external force. Spindle orientation appears to be a consequence of cell elongation along the zero-force direction in response to the dynamic shear. This process is a nonlinear response to the strain amplitude, requires actomyosin activity and correlates with redistribution of myosin II. Mechanosteered cells divide normally, suggesting that this mechanism is compatible with biological functions.

## Introduction

In mitosis, the orientation of the mitotic spindle determines the plane of cell division [1], [2]. If cell-type determinants are differentially located along the spindle axis, either as a consequence of intracellular polarity or of asymmetric external cues, then, as a consequence, the daughter cells will attain different fates [3]. External asymmetries may be given for example by morphogen concentration gradients, spatial variation in cell phenotype, or by the presence of tissue boundaries. The crucial role of such asymmetric cell divisions in the development of multicellular organisms has been revealed in many invertebrate and vertebrate systems [3]–[5]. They are also important in adult organisms, for example in skin stratification, where polarized basal cells dividing perpendicularly to the basal membrane generate a suprabasal daughter that differentiates and forms the skin barrier [6].

The simplest cue which determines spindle orientation is cell shape. In general, cells divide along their long axis [7]. Spindle orientation determined by cell shape seems to be sufficient to explain cell fate diversity in the Xenopus blastula, where fate-determining cell divisions perpendicular to the surface of the embryo correlate with a perpendicular long axis [4]. The positioning and orientation of the spindle is attained by an intricate, yet poorly understood balance of mechanical forces. Dynein motors, known to generate forces between the actin cortex and the astral microtubules that radiate away from the spindle, are widely believed to control spindle positioning [8], [9] and to generate spindle oscillations [10], [11]. By changing cell shape with a micropipette, O'Connell and Wang showed that the spindle monitors and reacts to externally imposed changes in cell shape [8]. In many systems, however, specialized biochemical cues are believed to override the cell shape cues [12]. This has been explored in vitro using fibronectin-coated patterns. As HeLa cells round up prior to mitosis, retraction fibers are formed that connect the cytoskeleton to the substrate; spindle orientation is then dictated by the fibronectin pattern rather than by cell shape [13], [14]. Recently, it has been shown that stretching such fibronectin-coated substrates induces spindle orientation along the direction of the external force [15]. Therefore, there seem to be two mechanisms by which external forces can influence the orientation of the division axis: by changes in cell shape, or via mechanosensitive responses elicited at specific adhesion points. It is an open question how cells integrate these mechanical cues; they may act synergistically or antagonistically. The answer may crucially depend on the geometry and timescale of the mechanical stimulation as well as on the adhesive conditions.

Here, we introduce shear deformations as a novel way to mechanically stimulate mitotic cells. Though not as common a method in experimental biomechanics as stretch or compression, shear strain is actually ubiquitous since it is present in any volume-preserving deformation. The only way to avoid shear is to perform a pure dilatation, that is, a uniform scaling in all directions; the majority of physiological strains obviously do not fall in this category and thus cells embedded in a strained tissue will undergo shear to some extent. The essential difference between our approach and more conventional ones is, rather, the spatial location of adhesion points. In our case, the parallel plates provide a 3D environment confining the cell. This stands in contrast to conventional 2D approaches where cells adhere on a single, flat substrate [15] - an important distinction since the geometry of the extracellular environment can radically change cell behavior [16]. Our experiment can be seen as a simple realization of a dynamic three-dimensional environment.

Using dynamic shear, we show that mitotic RPE1 and MC3T3 cell divide perpendicular to the external force. The orientation of the division axis appears to be a consequence of cell elongation in response to the external forces. This elongation process is mechanically nonlinear, actomyosin-driven, and of a remarkable efficiency: frequencies as slow as 30 mHz fully bias cell division. Immunofluorescence imaging of myosin II reveals a depletion of myosin in the equator relative to the poles of elongated cells, suggesting that the external forces drive actin cortex remodelling. Finally, mechanosteered cells divide normally, indicating that this phenomenon could have a biological function, and that it may be potentially exploited to guide in-vitro growth of artificial tissues.

## Results

### A novel approach to impose external forces on mitotic cells

**Figure 1:**
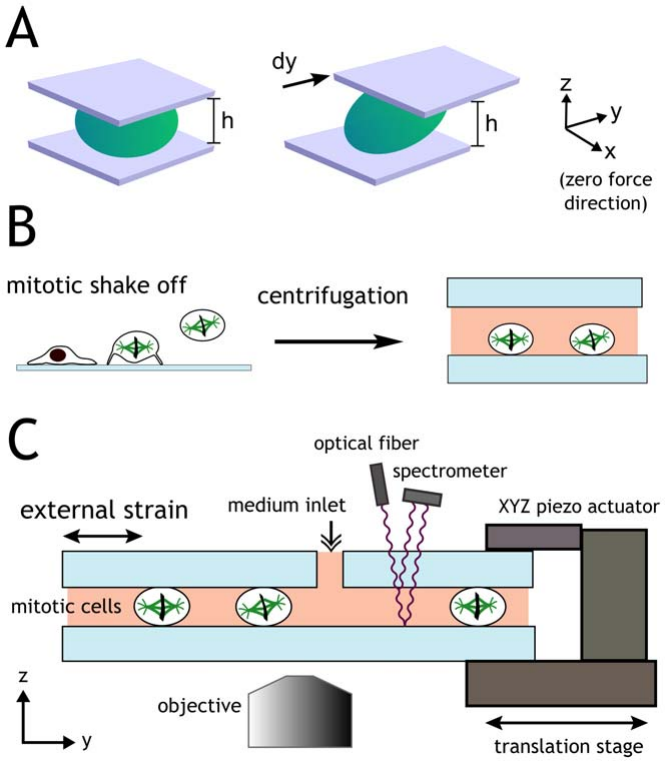
Experimental setup. **A:** Shear deformations are used throughout this work to mechanically stimulate cells. Cells are located between two flat substrates separated by a gap h. Cells are sheared by translating one substrate over a distance dy along the strain direction y (keeping the gap constant). The magnitude of the shear strain is given by γ  = dy/h. This geometry does not impose forces along x (the zero-force direction) and keeps cell volume constant. **B:** Mitotic cells are first harvested by shaking the culture flask, then collected by centrifugation, introduced in the chamber, and allowed to sink onto the bottom plate. Finally the top plate is brought down slowly. As soon as the final gap is reached, the temperature is increased from 25°C to 37°C. **C:** Experimental set-up. A monolayer of about 1000 cells is sheared between the two glass plates. The gap between the plates can be measured with micrometer accuracy by recording interference fringes with a hand-spectrometer. A flow of fresh medium ensures constant pH and oxygen supply. Coupled with an XY translation stage, the setup can collect data over hundreds of cells.

In order to assess the role of mechanical forces as a spatial cue for cell division, one needs to impose a force without giving an adhesion cue. At the same time it is necessary to exert large forces to strain the mitotic cells, which are stiff and poorly adherent [17]. To achieve these contrasting requirements, we designed an experimental set up that imposes shear forces on cells lying between two uniform substrates (Fig. 1A). While effectively confining the cell within a three-dimensional environment, this approach provides a two-dimensional realization of a purely mechanical spatial cue, because the only asymmetry within the plane of the substrates is given by the translation of one of the plates (Fig. 1A). An important feature of the used geometry is that no force is imposed in the direction perpendicular to the shear plane (Fig. 1A); we will refer to this direction as the “zero-force direction”. Advantageously, shear forces do not require strong adhesion (crucial for mitotic cells) [18] and they do not impose volume changes per se, which would possibly alter intracellular concentrations or intracellular pressure during the deformation.

Prior to an experiment, cells were harvested by mitotic shake-off, harvested by centrifugation and re-seeded on the bottom glass plate at room temperature. The cells were allowed to sink for 5 min (Fig. 1B). As soon as cells were lying on the bottom plate, the top plate was brought down slowly in order to minimize convective dispersion of the cells. With this procedure, cells do not form the retraction fibers seen in mitotic cells that had been plated well before mitosis onset on fibronectin surfaces [13]. In all experiments shown here, cells were gently compressed (20-30%) between the two glass substrates at a gap h = 10 µm (Fig. 1C). The gap can be measured with micrometer accuracy by exploiting the interference fringes between the two water-glass interfaces. For this, we used a fiber optic spectrometer to measure reflected light intensity as a function of wavelength λ (Fig. 1C). For normal incidence, the signal is a periodic function of the wavenumber 2π/λ with period 2h n_w_, where n_w_ = 1.33 is the refractive index of the aqueous medium. As soon as the final gap **h** was established, the temperature was raised to 37°C and the perfusing medium flow was switched on to provide oxygen and keep constant pH throughout the experiment. Dynamic shear force is applied to the cells by translating one of the substrates in a sinusoidal fashion with a given amplitude **dy**, which corresponds to a shear strain **γ = dy/h**. Experiments were performed using shear strain in the range 10%–100% and frequency in the range 0.01 Hz–30 Hz. Since mitosis is relatively slow, data from over hundred cells can be simultaneously collected by placing the set up on a translation stage and taking images subsequently on multiple different spots (Fig. 1C).

### Sheared mitotic cells elongate along the zero-force direction in an actomyosin-dependent manner

**Figure 2:**
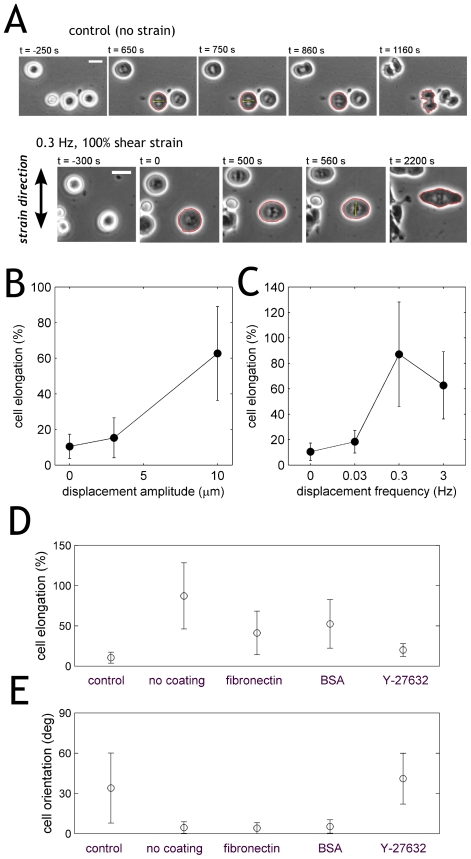
Sheared mitotic cells elongate along the zero-force direction in an actomyosin-dependent manner. **A:** Non-strained cells divide isotropically. Strained RPE1 cells elongate and divide along the zero-force direction. **B:** RPE1 cell elongation before anaphase onset as a function of amplitude for a constant frequency 3 Hz. **C:** elongation as a function of frequency for a constant amplitude (100%). Glass plates were not coated in A,B,C. **D:** Cell elongation is also observed with albumin-passivated as well as with fibronectin-coated glass plates. ROCK inhibition by Y-27632 at a concentration of 10 µM abolishes cell elongation. **E:** Cell orientation for all plate coatings and ROCK inhibition.

Under application of dynamic shear strain, mitotic RPE1 cells show a well-defined change in morpohology: they markedly elongate along the zero-force direction, i.e. perpendicular to the plane of shear strain (Fig. 2A, see also Supplementary Movie S1). To quantify the cell elongation, we tracked the cell contour and calculated the ratio R of the major and minor axes of the ellipse with the same second moments as the cell body [19]. This procedure is valid because the shapes of metaphase cells were close to elliptic under all experimental conditions. For a more intuitive measure of cell shape, we define “cell elongation” as (R-1)×100%, where a round cell has an elongation of 0, whereas a cell whose major axis is twice its minor axis has 100% elongation (i.e., (2–1)x100%). Non-strained metaphase cells typically have elongations of 10% (Fig. 2B, zero amplitude). Imposing shear strain induces cell elongation in a nonlinear fashion (Fig. 2B). Varying the frequency of the stimulation reveals a time-dependence of the response: cell elongation is stronger for frequencies larger than 100 mHz (Fig. 2C). We also noticed that the shear-induced cell elongation can be observed on substrates passivated with albumin as well as on substrates coated with fibronectin, although in both cases the extent of elongation was slightly less than on the non-coated substrates (Fig. 2D,E).

Since cell shape is mostly determined by the actin cortex, we inhibited myosin activity with the ROCK (Rho-associated coiled kinase, a main regulator of the actin cytoskeleton) inhibitor Y-27632 at a concentration of 10 µM, which reduces actin cortex tension by approximately 50% in L929 fibroblasts [20]. Indeed, under these condition the elongation of mitotic cells was diminished upon application of shear strain (Fig. 2D,E).

### Shear-elongation by 15% suffices to align the mitotic spindle

**Figure 3:**
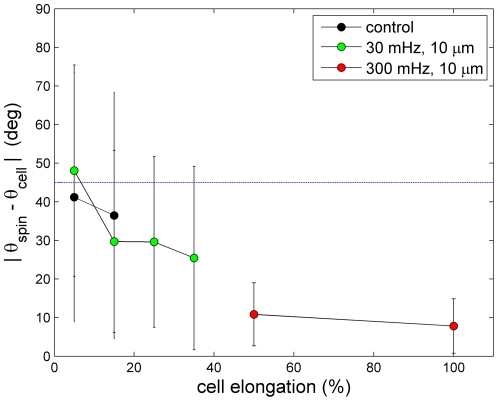
Shear-elongation by 15% suffices to align the mitotic spindle. Angular difference between the mitotic spindle axis and the major axis of the cell body as a function of cell elongation. Black: non-sheared cells. Green: low frequency 0.03 Hz, large amplitude 100% shear strain. Red: faster frequency 0.3 Hz, large amplitude 100% shear strain. Cells were RPE1 and the glass plates were not coated.

Cell shape is a major determinant of the division axis: in the absence of other adhesive cues cells generally align the mitotic spindle along their long axis [8]. Therefore we asked whether the cell elongation induced by the external shear strain can bias the orientation of the mitotic spindle. For this we measured angular position both of the cell body θ_cell_ and the mitotic spindle θ_spin_, and plotted the difference in angles [θ_cell_ - θ_spin_] as a function of the cell body elongation. As shown in Fig. 3, cell elongation below 10% did not lead to a significant bias: spindles were positioned randomly relative to the cell axis, both in control, non-sheared cells as well as in cells sheared at a low frequency (0.03 Hz). However, already at elongations extending 10% there was a significant bias of the spindle towards the long axis of the cell body. Increasing the frequency of the mechanical stimulation to 0.3 Hz drastically increased the extent of cell elongation and completely biased spindles towards the zero-force direction (Fig. 3). Thus, the mitotic spindle is very sensitive to the aspect ratio of the cell, which is in a good agreement with a recent study performed under static conditions [21].

### The mitotic spindle adapts to time-varying external forces via actomyosin activity

**Figure 4:**
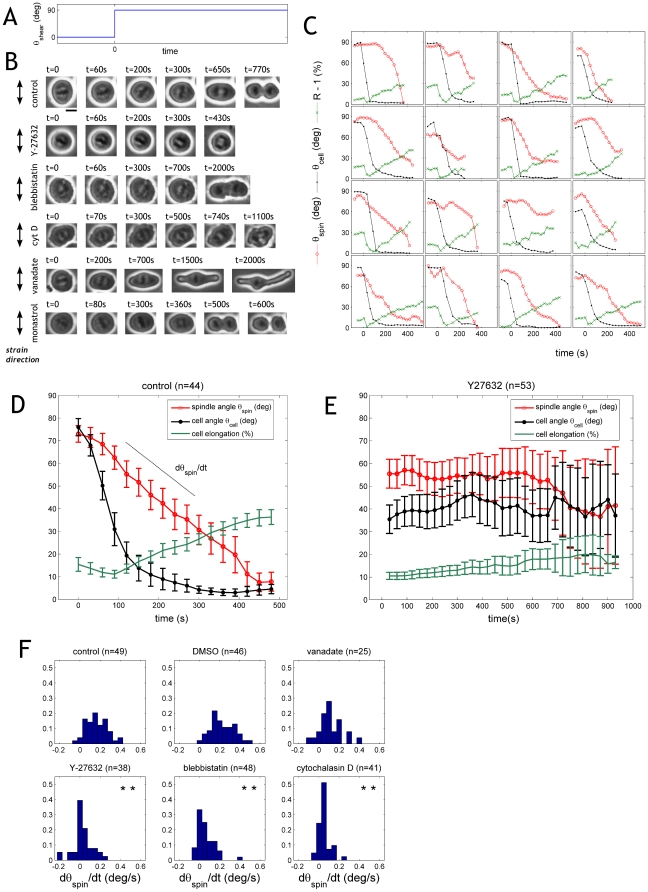
The spindle adapts to time-varying forces via actomyosin. **A:** Immediately after increasing the temperature to 37°C, RPE1 cells were stimulated by application of shear force along the X axis at frequency 0.3 Hz and strain amplitude 100%. After 10 min, at time t = 0, the direction of the force was changed by 90 deg from X to Y and the subsequent response of cell shape and spindle orientation was analyzed. **B:** Immediately after rotation of the external force, control (non-treated) cells became round and subsequently elongated along X. Simultaneously, the mitotic spindle rotated from Y to X. In contrast, cells treated with the ROCK inhibitor Y-27632 neither elongated nor re-oriented the mitotic spindle. Similarly, cells treated with blebbistatin or cytochalasin D did not rotate the spindle (cell shape is ill-defined under these conditions). In contrast, cells treated with vanadate (300 µM) rotated the spindle away from the strain at the same pace as non-treated cells, but often failed to separate chromatides and the spindle collapsed. Note that the cell elongation reached abnormal values, likely due due to the longer time spent in metaphase. **C:** Spindle angular position (red), cell major axis angular position (black) and cell elongation (green) as a function of time, for a representative subset of 16 non-treated cells. Curves terminate at anaphase onset. **D:** Average values of 44 cells as a function of time. Data is shown as mean +/− standard deviation. **E:** Cells treated with Y27632 at a concentration of 10 µM. **F:** Distribution of spindle rotation speed for different drug treatments. Negative rotation speeds correspond to spindle rotating towards the external strain. Statistical significance relative to the control was assessed using a Mann-Whitney U-test. One asterisk indicates P<0.01, two asterisks indicate P<10^−8^. Actomyosin inhibition by either Y-27632, blebbistatin, or cytochalasin D all led to significantly slower rotation rates as well as loss of directionality (negative rotation rates). In contrast, vanadate did not have a significant effect on spindle rotation.

To further assess the causal relationship between cell shape and spindle orientation, we examined whether and how they both react when the direction of the external force suddenly changes. To this end, we stimulated the RPE1 cells for 8 min along the direction X and then switched to the perpendicular direction Y (Fig. 4A). In an immediate response to the change of the strain-direction, the cells became round (Fig. 4B, control; notice also the decrease in the average cell elongation in Fig. 4,D); then, within 5 min, 95% of cells elongated along the new zero-force direction. Simultaneously with the elongation, the cells rotated their spindles away from the strain (Figs. 4C,D; see also Supplementary Movie S2).

To identify the underlying molecular machinery, we investigated the effect of biochemical perturbation on spindle re-orientation. For this experiment it is crucial to avoid disruption of the mitotic system; thus we used chemical inhibitors that did not interfere with spindle bipolarity. The ROCK-inhibitor Y-27632 had no significant effect on spindle morphology in RPE1 cells (see Supp. Mat. Fig. S2). We observed that ROCK inhibition fully abolishes spindle rotation as well as cell elongation (Fig. 4E). Note that cortical tension is not completely lost under these conditions as the mitotic cells maintain their smooth, round shape (Fig. 4B). Accordingly, similar results were obtained with the myosin II inhibitor blebbistatin (50 µM) and with the potent actin polymerization inhibitor cytochalasin D (10 µM); both lead to a significant slow down of the rate of spindle re-orientation, which in many cells manifested as a complete halt (Fig. 4B,F).

Taken together, these results show that mitotic cells actively respond to external shear forces. The actin cortex reshapes within a few minutes after a change in the external force direction. The mitotic spindle follows cell shape instantaneously (within our time resolution of ∼20 s), indicating that the machinery of spindle orientation must be faster than the actin cortex remodelling.

An alternative hypothesis to explain the observed spindle rotation is that the external forces have a direct effect on the motor proteins that are responsible for positioning of the spindle. For example dynein has been shown to control spindle positioning [8], [9] and to generate spindle oscillations [10], [11]. If we assume that the external force may detach these motor proteins, then the spindle should rotate away from the force with a rotation speed that depends on dynein motor activity. To test this alternative explanation, we treated the cells with orthovanadate at a concentration of 0.3 mM. At this concentration orthovanadate inhibits dynein but not kinesins or myosin in HeLa cells [22] and no significant alterations of spindle morphology can be observed [8] (see Supp. Mat. Fig. S5 for the effect on RPE1 cells). Subsequently, we measured the speed of spindle rotation after a sudden change in the direction of the external force. We observed that the time required for cell division was significantly prolonged (Supp. Mat. Fig. S6), consistent with the observation that microinjection of anti-dynein antibody or p50 dynamitin induces metaphase arrest [23]. However, we did not observe a significant change in the spindle rotation rates (Fig. 4F). This suggests that dyneins are not crucial for spindle re-orientation under the conditions of our experiment.

### Sheared cells have more myosin II at the poles than at the equator

**Figure 5:**
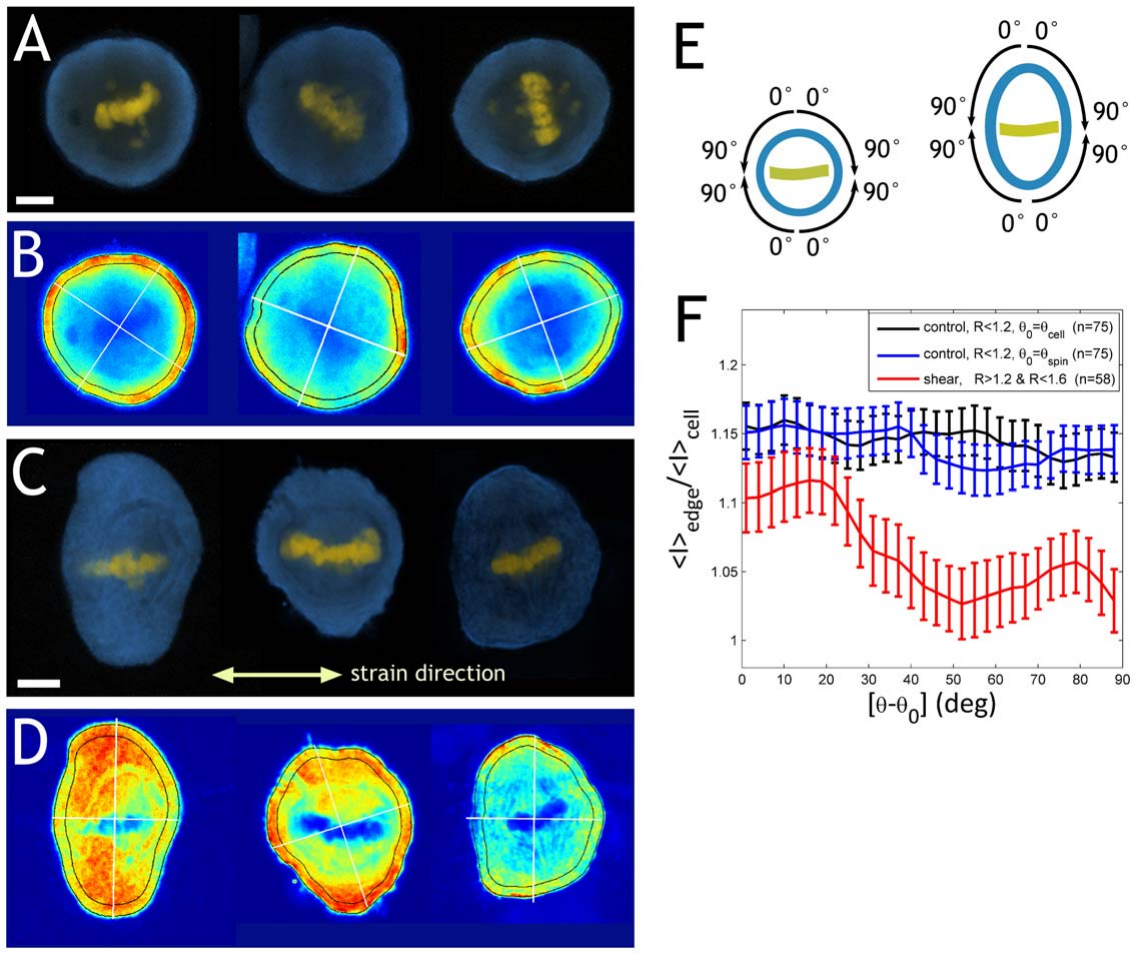
Shear-elongated cells have more myosin at the poles. **A:** Immunofluorescence images of control (non-sheared) RPE1 cells. Blue is myosin II, yellow is DNA stain. Bar: 5 µm. **B:** color-coded intensity of myosin stain for the cells shown in A. The white lines mark the position of the major and minor axis as recognized by the algorithm. The outlined perifery region was used to quantify myosin intensity (see fig.F). **C:** immunofluorescence images of sheared RPE1 cells (amplitude 100% strain, frequency 0.3 Hz). Bar: 5 µm. **D:** color-coded myosin intensity of the cells shown in C. **E:** for statistics, we averaged the angular distribution of myosin intensity over several cells (control: n = 75, sheared: n = 58). Since there are no signs of polarity or chirality in the images, we split each cell into four quadrants and averaged the data over them. For the reference angle θ_0_ we considered two possibilities, the cell body orientation and the chromosome plate orientation. **F:** mean myosin signal intensity throughout the perifery as a function of angle θ-θ_0_, normalized by the mean intensity over the whole cell body. In the case of the control cells, we analyzed the results using two different reference angles: the orientation of the cell body (θ_0_ = θ_cell_, **black curve**) and the orientation of the spindle (θ_0_ = θ_spin_, **blue curve**). For sheared cells (**red curve**) this distinction play no role, since the difference between the two orientations is at most 20 deg. Normalized intensities were averaged over multiple cells, as well as over the four quadrants of each cell by reflecting about θ_0_ and θ_0_ + 90 deg (see E). Error bars denote +/− 2 S.E. Control cells showed an essentially flat signal, regardless whether we took the cell body or the spindle as reference. In contrast, sheared cells showed more myosin intensity around the poles (θ = θ_0_) than close to the equatorial plane.

We have shown that actomyosin is necessary for mitotic cell elongation perpendicular to the external forces. One possible mechanism underlying this phenomenon could be a redistribution of myosin induced by the external forces. We explored this possibility by means of immunofluorescence confocal microscopy. For this, we fixed shear-elongated RPE1 cells as well as non-sheared control ones and stained them using standard protocols (see material and methods). To avoid premature progression to anaphase with formation of a contractile ring, we treated cells with the proteasome inhibitor MG-132. This treatment arrests cells in metaphase without a significant alteration of spindle morphology (see Supp. Mat. Fig. S7). In control cells, myosin II was evenly distributed within a ∼ 2 µm wide strip along the edge of the cell (Fig. 5A,B). In contrast, shear-elongated cells showed more heterogeneity in the myosin II signal, with significant amounts of myosin in the region of the mitotic spindle (Fig. 5C,D).

To precisely examine the changes in the intensity and distribution of the myosin signal, we undertook a quantitative image analysis. Since cell shape is determined by the actin cortex [20], we quantified myosin signal intensity in a 1 µm wide perifery region at the cell edge (see Figs. 5B,D). To average over multiple cells, we defined a reference angle θ_0_ for each individual cell. Moderately elongated cells have a well-defined unique axis since the body axis is parallel to the spindle axis within 20 deg (Fig. 3). However, round (control) cells often have an ill-defined body orientation θ_cell_ that depends on the image analysis algorithm. Therefore, we analysed the data using as angular reference the orientation of the spindle axis θ_spin,_ defined as the direction perpendicular to the metaphase chromosome plate. We split each cell into 4 quadrants starting from θ = 0 (the cell poles) to θ = 90° (the position of the chromosome plate, see Fig. 5E) and pooled the data from each quadrant. In order to minimize artefacts possibly arising from extremely elongated cells, we only considered cells with elongations below 60%; the average elongation of sheared cells considered in the analysis was 32%±5%, whereas that of control cells was 10%±1%. As shown in Fig. 5F, control cells did not show a significant angular dependence of myosin signal intensity, regardless whether the cell body or the spindle was used as angular reference. In contrast, shear-elongated cells had significantly more myosin at the poles than at the equator. This indicates that external forces induce myosin II redistribution, which in turn may be the causal factor leading to cell elongation.

### Shear forces orient the division axis without compromising mitosis

The observations described above concern the behavior of cells in the earlier stages of mitosis, prophase and metaphase. Further progression through mitosis requires proper assembly of the mitotic spindle, thus ensuring error-free segregation of genetic material. Otherwise, activation of the spindle assembly checkpoint will delay anaphase onset for up to many hours [24]. Thus we asked whether cells divide properly in the conditions of our experiment.

**Figure 6:**
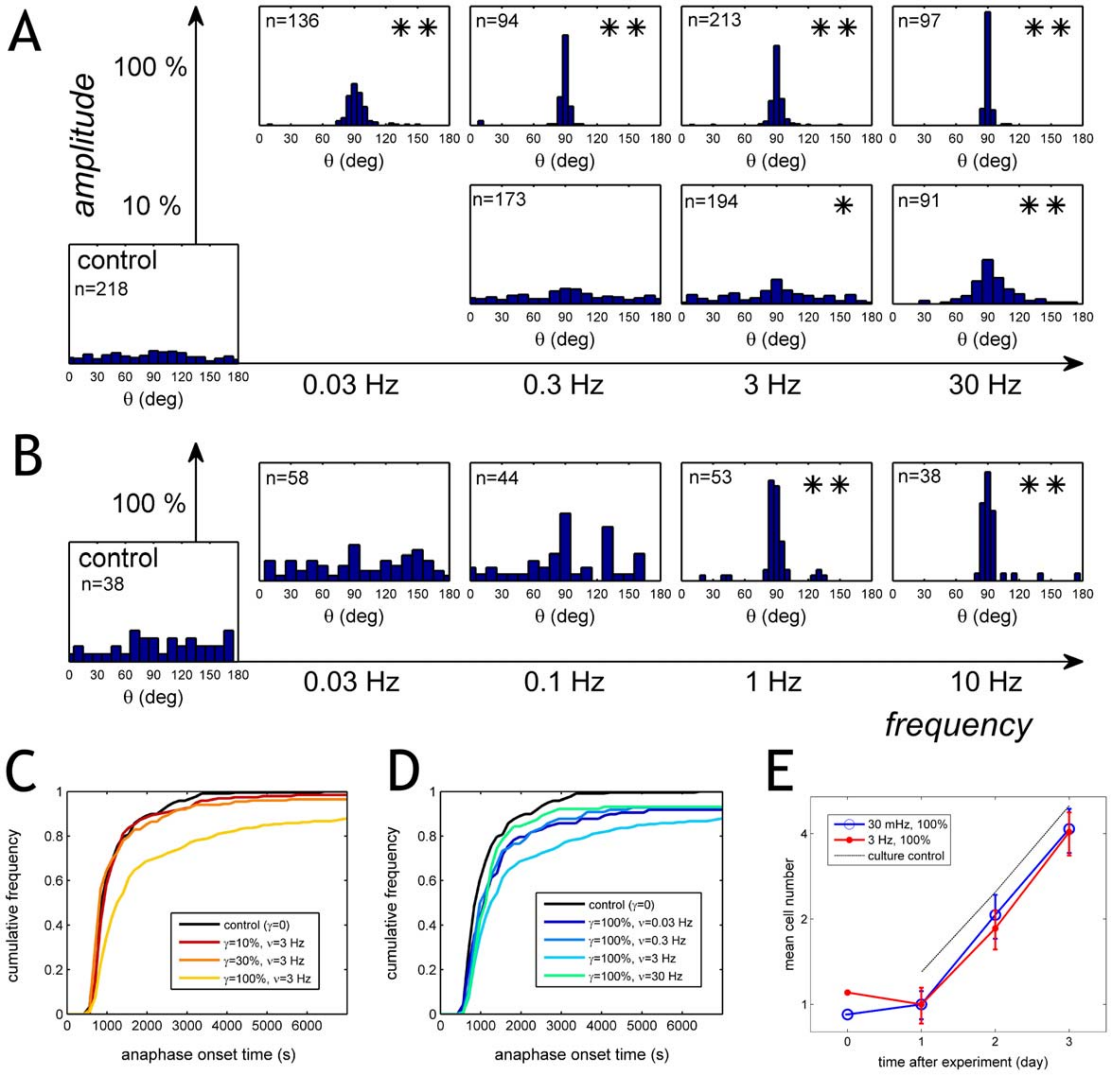
Shear forces orient the division axis without compromising mitosis. **A:** Division angle distributions for different amplitudes (vertical axis on the left) and frequencies (horizontal axis at the bottom) for RPE1 cells. At small amplitudes (γ = 10%), anisotropic division requires a very high frequency (30 Hz). At large amplitudes (γ = 100%), even very slow stimulations (0.03 Hz) suffice to align the division axis in essentially all cells. Statistical significance relative to the control was assessed using a Mann-Whitney U-test on the reflected angles | θ - 90° |. One asterisk indicates P<0.01, two asterisks indicate P<10^−8^. **B:** Similar results were obtained with the osteoblast cell line MC3T3. **C:** Cumulative histograms of anaphase onset times in the presence of dynamic forces for RPE1 cells. Each curve is a different experiment, performed at varying amplitudes and the same frequency 3 Hz. **D:** Cumulative histograms of anaphase onset times of RPE1 cells for different frequencies and constant large amplitude 100%. Strain-alignment of the division axis (which is observed for all frequencies at this amplitude) delays division only marginally. A significant delay is observed at 3 Hz; note that cell division becomes again fast at 30 Hz. **E:** RPE1 cell growth after force-alignment of the spindle. After each experiment, cells were collected, plated on a gridded dish (Ibidi, Munich, Germany) and the growth of individual cells was followed over three days. Note that cells are synchronized by shake-off prior to the experiment and re-seeded afterwards. After one day, cells grow exponentially. To ease comparison of the exponential growth phase between different treatments, we divided cell number by its value at day 1. For a control, we followed also the growth of cells that had been in normal culture conditions throughout (black dashed line). No significant difference in growth rate can be observed after mechanical stimulation at 0.03 Hz, 100%. Nevertheless, we observed increased cell death within the first day following stimulation at 3 Hz, 100%, probably indicating damage by the non-physiological stimulation.

First, we assessed the ability of cells to divide in static conditions. We performed a control experiment with RPE1 cells gently compressed between the two static glass plates, without applying shear forces. We observed that 99% of mitotic cells (n = 218) successfully divided under these conditions. As expected, cells divided isotropically; the division axis was uniformly distributed within the plane of the substrate surface (Fig. 6A, control). Next, we imposed dynamic forces and followed the fate of individual cells during two hours. As expected from our previous observations, RPE1 cells aligned their mitotic spindle and divided perpendicularly to the strain direction, along the zero-force direction (Fig. 6A; see also Supplementary Movie S1). Most cells that were initially in prophase assembled their spindles along the zero-force direction. Overall, the alignment process has a remarkable efficiency at large amplitudes, as shown by the sharply peaked distributions in Fig. 6A. At the large shear amplitude 100% and a slow frequency 0.03 Hz, 92% of all mitotic cells (n = 136) achieved successful division; 90% of them divided perpendicular to the force within a 30 deg window. Increasing the frequency to 0.3 Hz narrowed this window to a width of 10 deg (Fig. 6A).

Identical behavior was observed using the osteoblast cell line MC3T3-E1. Again, the cells divided perpendicularly to the external force, for all tested frequencies from about 0.3 Hz up to 10 Hz and large amplitude (100%) (Fig. 6B).

Since the spindle assembly checkpoint is believed to rely on mechanical cues [24], [25], one may expect that external forces would interfere with the progress from metaphase to anaphase. We therefore asked whether imposing dynamic forces influences the efficiency and timing of the RPE1 cell division. An experimentally well-defined measure of division time is given by the time elapsed from the beginning of the experiment until anaphase onset. The initial time is when the experiment temperature is increased from 25°C to 37°C; the anaphase onset time is defined by the separation of the chromatides, a fast process which could be resolved in time within 20 s. Most of the experimental conditions did not affect the time of anaphase onset, but there was a significant delay specifically at the large amplitude 100% and the intermediate frequency 3 Hz (Fig. 6C,D). Nevertheless, even in these conditions, 88% of the RPE1 cells divided within two hours (n = 213).

To learn how re-orientation of the spindle alters the viability of the cells, we followed cell survival for several days after the application of external forces. The RPE1 cells were collected immediately after the experiments, re-plated into the fresh medium and we followed their growth for over three days. Cells that had been oriented during mitosis by application of large strain (100%) at a slow frequency (0.03 Hz) grew at a rate indistinguishable from the control (Fig. 6E), indicating that no extensive cell death took place either due to the strain or due to chromosome missegregation. Thus, although cells under the applied strain undergo spindle re-orientation and, in some instances, delay the onset of anaphase, their ability to successfully divide and further propagate remains unimpaired. Note that excessive strain cycles can irreversibly damage the cells, as documented by the observance of slightly slower growth after mechanical perturbation at the fast frequency of 3 Hz (Fig. 6E).

## Discussion

Here, we show that mitotic eukaryotic cells under dynamic shear divide perpendicular to the external force, i.e., along the zero-force direction that is perpendicular to the shear plane. We observed a similar behavior in both human RPE1 epithelial cells and mouse MC3T3 osteoblasts. The orientation of the division axis seems to be a consequence of the fact that sheared mitotic cells elongated along the zero-force direction. This cell elongation is actomyosin-dependent since it could be fully abolished by blocking myosin or ROCK activity. We observed a remarkable sensitivity of the mitotic spindle to the cell shape: aspect ratios as small as 1.2 were enough to bias the mitotic spindle towards the long axis in RPE1 cells. This is in good agreement with a recent study where cell shape was changed by static means [21]. The dramatic cell elongation induced by shear forces at high frequencies explains their remarkable efficiency to bias cell division.

Why do mitotic cells under shear elongate away from the external force? There are two parts to this question: i) what determines cell shape, and ii) how the external forces alter it. Cell shape is in general determined by the cytoskeleton, and in loosely adherent mitotic cells the essential component is probably the actin cortex [17]. The relation between actin cortex mean curvature **C**, membrane tension **σ** and pressure difference **Δp** is given by the Laplace law, **Δp = σC** . In our case we expect the hydrostatic pressure to be uniform since the oscillation period is rather long compared to pressure equilibration times. Thus, in an elongated cell, the spatial variation of the cortical curvature should correspond to gradients in the active forces exerted by the actomyosin system. Force gradients may result from different sources, such as variations in cortical density or thickness, or in myosin activity. Analysis of the myosin II immunofluorescence experiments revealed that the signal is stronger by approximately 10% at the poles than at the equator of elongated mitotic cells. This result seems difficult to reconcile with the simple view of a Laplace law with constant pressure. If one considers only the curvature in the x-y plane, then one would expect to find considerably more myosin at the equator. Since the ratio of the maximal and minimal curvatures of an ellipse goes as the third power of the axes ratio, the curvature Cp at the pole of a 30% elongated cell would be about twice as much as the curvature Ce at the equator, Cp = (1.3)^3^ Ce ∼ 2 Ce. This suggests that myosin signal intensity alone cannot explain the elongated cell shape, although one needs to keep in mind that we cannot accurately measure the curvature along z (perpendicular to the substrate). We thus propose that either myosin activity or actin cortex structure (or both) change along the cell contour.

How do the external forces achieve cell elongation? One may speculate that mechanosensing responses are involved. In mitotic Dictyostelium cells, a mechanosensory system controls cell shape during anaphase via myosin-II recruitment, seemingly to ensure equal-sized daughters [26]. On the other hand, the response to the mechanical stimuli in our cells is non-linear (Fig. 2B), which is consistent with a mechanical restructuring of the actin cortex, possibly similar to the remodelling of bundled actin networks [27]. Resolving this question would require systematic knockdown of mechanosensing pathways as well as live microscopy of the actomyosin system, a task left for future work.

Interestingly, recent in-vitro experiments performed on mitotic cells adhering on single flexible substrates have revealed cell division parallel to the external force [15], rather than perpendicular as in our case. Fink et al performed experiments on cells adhering to fibronectin-coated substrates via retraction fibers, which seem to be very effective in transducing external strain to the cell body. The fibronectin patterns were designed in such a way as to keep cells round during spindle rotation [15]. In our case, by detaching mitotic cells before introducing them into the chamber, retraction fibers were absent during the experiment: accordingly cells underwent extensive shape remodeling. Furthermore, whereas in Fink et al the cell shape was changed stepwise from an elongated shape to a round one, we applied a steady oscillatory stimulus. Thus, the apparent discrepancy is likely caused by the differences in adhesive conditions and mechanical stimulus.

Taken together, we have shown that mitotic RPE1 cells under dynamic shear undergo actomyosin-driven elongation and division along the direction perpendicular to the external forces. Our observation of diminished myosin II immunofluorescence signal at the equator of elongated cells suggests that the external strain induces gradients in actomyosin structure. Further work will be required to resolve whether this follows from purely mechanical remodelling or from more complicated mechanosensing responses. Our findings provide a novel approach to study the intricate connections between the mitotic apparatus, the actomyosin system and the extracellular environment.

## Materials and Methods

### Experimental set up

The set up is a micrometer-sized shear chamber based on the Cell Monolayer Rheology technique [18]. A monolayer of cells is squeezed and sheared between two glass substrates, which allows for large scale mechanical stimulation of cells independently of the adhesive conditions. Glass substrates are optically flat BK7 glass windows (Edmund Optics GmbH, Karlsruhe, Germany). Substrate alignment is done by exploiting interference fringes between the two substrates: if only one fringe can be observed across the substrates, then these are parallel within a micrometer. Interference can also be used to accurately measure the gap between the substrates (Fig. 1C). The intensity of light reflected from two parallel plates is a periodic function of the wavenumber 2π/λ with period 2h n_w_, where h is the gap and n_w_ = 1.33 is the refractive index of the aqueous medium. We measured the wavelength dependence of the reflected light intensity using a hand spectrometer (Laser 2000, Wessling, Germany). Substrate translation (necessary to compress and shear cells) is achieved using piezoelectric translators (Physik Instrumente, Germany). A peristaltic pump (Ismatec, Switzerland) drives a steady flow of medium through a hole drilled in the top plate (Fig. 1C).

#### Cell culture

We use hTERT-immortalized RPE1 cells as well as mouse MC3T3-E1 osteoblasts. Both are grown in DMEM medium (PAA, Austria) with 10% FBS (PAA) and supplemented with penicillin-streptomycin. The cells were splitted every two days and used between passages 4 and 18.

### Experimental procedure

Mitotic cells were harvested by mitotic shake-off. The cells were splitted 24 hrs before the experiment to ensure 30% cell confluency for every experiment. After harvest, cells were centrifugated in an Eppendorf tube at 7000 rpm for 2 min and resuspended in PBS at 10°C. The cell suspension was transferred to the gap between the glass substrates and the top substrate brought down until the cells got gently squeezed at a gap of approximately 10 µm. Then, at time t = 0, the temperature was raised to 37°C and medium perfusion and oscillatory strain were turned on. Medium perfusion at a rate of 1 µl/min ensures supply of oxygen and nutrients and prevents pH changes by evaporation of CO_2_. This rate corresponds to a flow velocity of about 50 µm/s in the vicinity of the analyzed cells.

### Biochemical perturbation experiments

We performed biochemical perturbation experiments in order to assess the role of different cytoskeletal components. For this we used cytochalasin-D (Tocris Biosciences, Bristol, UK) orthovanadate, Y-27632 and blebbistatin (Sigma-Aldrich). Prior to biochemical perturbation experiments, cells were incubated in their regular culture flasks with inhibitory drugs (vanadate, 1 h 30 min; blebbistatin, 30 min; Y-27632, 30 min; DMSO, 1 h 30 min) in order to ensure drug effect. Since cytochalasin-D induces rapid cell detachment, a previous incubation step was not performed as it would have been incompatible with subsequent harvest by shake-off. In this case, however, the drug effect could be easily corroborated (Fig. 4B). All drugs were present in the perfusing medium throughout experiments. Since blebbistatin, monastrol, cytochalasin-D and nocodazole are stock-diluted in DMSO, control experiments were done with DMSO at a concentration of 0.1%, higher than in any of the drug-experiments. Drug concentrations were tuned so as to maximize cell perturbation without destroying bipolar spindle architecture. We had previously confirmed by fluorescence microscopy that RPE1 cells had majority of normal bipolar spindles under all conditions (Supp. Mat. Figs. S1, S2, S3, S4, S5).

### Image capture analysis

The images were taken with a 10X phase contrast objective and contain about 10 mitotic cells each. The initial cell locations are manually defined by the user; the image analysis program (a Matlab script) runs automatically until cytokinesis takes place. The edge of a round cell is very bright and can be easily detected by thresholding. To distinguish between the cell and other bright regions such as incomplete edges of neighbouring cells, the cell is defined as the largest hole in the image by means of standard topology-recognition Matlab routines. Since the shape of mitotic cells is in general close to elliptic, it can be characterized in terms of the ellipse with the same moments as the cell body. Cell elongation is defined as the ratio of major axis to minor axis, and cell orientation is defined as the angle of the major axis. The mitotic spindle is also found by thresholding. For this, additional constraints are necessary. The spindle is assumed to lie around the center of the cell (the centroid of the cell body) and to have a fixed size. Spindle angle is similarly defined as the angle of the major axis of the equivalent ellipse. See Fig. 2A for an example of the performance of the image analysis program.

### Immunofluorescence of sheared cells

Mitotic RPE1 cells were harvested by shake-off, centrifuged, and resuspended in medium with the proteasome inhibitor MG-132 (20 µM, Sigma). During the mechanical stimulation, the perfusing medium contained 20 µM MG-132.sm After stimulation for 40 min, cells were washed by perfusing PBS for 5 min, then fixed by perfusing 3% formaldehyde solution for 20 min, and finally washed again by perfusing PBS for 10 min. After fixation, the plates can be separated without straining the cells which detach from one of the plates and remain firmly attached to the other one. Cells were stained using a standard protocol. Briefly, cells were first treated with 50 mM NH_4_Cl for 20 min to passivate formaldehyde, washed with PBS, treated with 0.1% Triton X-100 for 3 min and washed with 2% BSA in PBS for 20 min; then incubated for 1 h with a primary antibody against myosin II non-muscle (Sigma M8064) diluted 1/50 in a PBS solution containing 2% BSA, washed with PBS, stained with a secondary antibody (Alexa Fluor 488 anti-rabbit IgG, Invitrogen A11008) and washed with PBS. For microscopy, the glass plate was covered with a drop of glycerol 90% and a cover slip, and finally imaged with a confocal microscope (Leica, Germany).

## Supporting Information

Movie S1 Force-alignment of the mitotic spindle. Imposing dynamic shear force at a frequency of 30 mHz to RPE1 cells leads to spindle alignment along the zero-force direction, i.e., perpendicular to the strain direction. Movie duration: 1 h 20 min.
(AVI)

Movie S2 Initially, RPE1 cells are strained along the horizontal direction. Then, the strain direction is stepwise changed by 90 deg. Within approximately 5 min, most cells adapt to the new stimulus by rounding up, re-elongating along the new zero-force direction, and rotating the mitotic spindle. Average data for this response can be seen in Fig. 4D. Movie duration: 1 h 5 min.
(AVI)

Figure S1 Cells were treated with DMSO. The concentration was 0.1%, higher than in any of the drug-experiments. The pictures show cells in prometaphase and metaphase (top row), anaphase (middle row) and late telophase (bottom row) stained for tubulin (left), actin (middle) and chromosomes by expression of H2B-GFP (right, overlay with DIC image). The observed cells do not show abnormalities throughout mitosis.
(TIFF)

Figure S2 Cells treated with the ROCK inhibitor Y-27632 in pro-/prometaphase (top row), meta- & telophase (middle row) and anaphase (bottom row) stained for tubulin (left), actin (middle) and chromosomes (right, overlay with DIC image). The microtubules occasionally look slightly longer than usual, but, since all mitotic stages are represented, the cells still undergo mitosis. The fact that a functional contractile ring assembles underscores the mildness of the treatment, which inhibits myosin indirectly as a result of ROCK inhibition.
(TIFF)

Figure S3 Cells treated with Blebbistatin in prophase (top row), metaphase (middle row) and anaphase (bottom row) stained for tubulin (left) actin (middle) and chromosomes (right column, ooverlay with DIC image). Functional bipolar spindles can be observed. Cells enter anaphase but, as expected for strong, direct inhibition of myosin, a contractile ring cannot form.
(TIFF)

Figure S4 Cells treated with the actin polymerisation inhibitor cytochalasin D. Staining was for tubulin (left column), actin (middle column) and chromosomes (right column, overlay with DIC image). The pictures clearly show the expected disturbance of the actin cortex as well as the inability of the cells to undergo cytokinesis.
(TIFF)

Figure S5 Cells treated with orthovanadate in prophase (top row), prometaphase (second row), metaphase & anaphase (third row) and telophase (bottom row) which had been treated with orthovanadate. Staining was for tubulin (left column), actin (middle column) and chromosomes (right column, overlay with DIC image). The different phases of mitosis appear normal.
(TIFF)

Figure S6 Orthovanadate treatment leads to a significant delay in anaphase onset.
(TIFF)

Figure S7 Uncompressed RPE1 cells stained for DNA using YO-PRO-1 (Invitrogen, Darmstadt, Germany) (left column) and tubulin (middle column). Right column: overlay, scale bar 5 µm. MG132 (Z-Leu-Leu, Sigma-Aldrich) is a proteasome inhibitor and prevents the mitotic cell from entering anaphase without affecting the mitotic spindle. These images are projections of a 3 µm z-stack around the cells centre obtained with a confocal microscope.
(TIFF)
