# Supplementary figures and images for: Mitotic Spindle Orients Perpendicular to the Forces Imposed by Dynamic Shear
Source: PLoS One. 2011 Dec 29;6(12):e28965. doi: 10.1371/journal.pone.0028965 (PMC3248423; doi:10.1371/journal.pone.0028965)

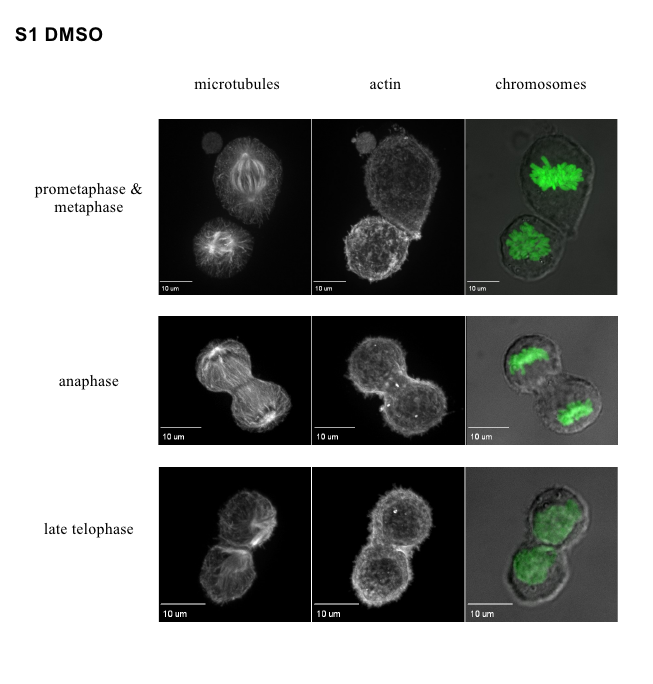

Supplement: Figure S1 — Cells were treated with DMSO. The concentration was 0.1%, higher than in any of the drug-experiments. The pictures show cells in prometaphase and metaphase (top row), anaphase (middle row) and late telophase (bottom row) stained for tubulin (left), actin (middle) and chromosomes by expression of H2B-GFP (right, overlay with DIC image). The observed cells do not show abnormalities throughout mitosis. (TIFF) [file pone.0028965.s003.tiff]

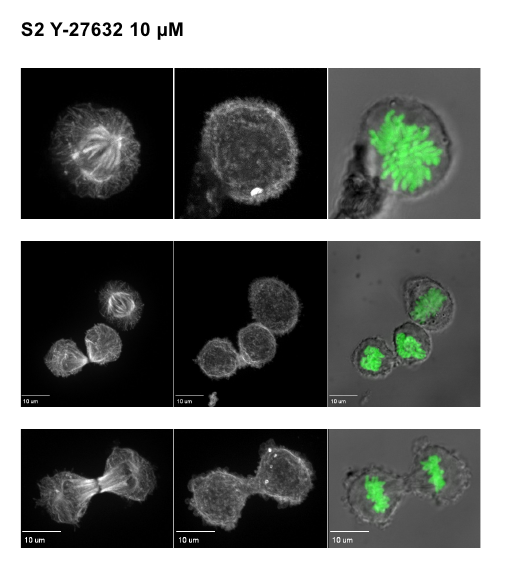

Supplement: Figure S2 — Cells treated with the ROCK inhibitor Y-27632 in pro-/prometaphase (top row), meta- & telophase (middle row) and anaphase (bottom row) stained for tubulin (left), actin (middle) and chromosomes (right, overlay with DIC image). The microtubules occasionally look slightly longer than usual, but, since all mitotic stages are represented, the cells still undergo mitosis. The fact that a functional contractile ring assembles underscores the mildness of the treatment, which inhibits myosin indirectly as a result of ROCK inhibition. (TIFF) [file pone.0028965.s004.tiff]

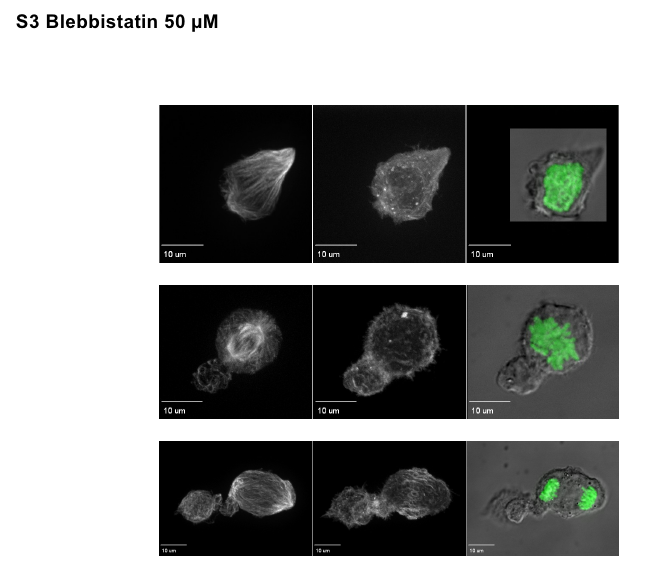

Supplement: Figure S3 — Cells treated with Blebbistatin in prophase (top row), metaphase (middle row) and anaphase (bottom row) stained for tubulin (left) actin (middle) and chromosomes (right column, ooverlay with DIC image). Functional bipolar spindles can be observed. Cells enter anaphase but, as expected for strong, direct inhibition of myosin, a contractile ring cannot form. (TIFF) [file pone.0028965.s005.tiff]

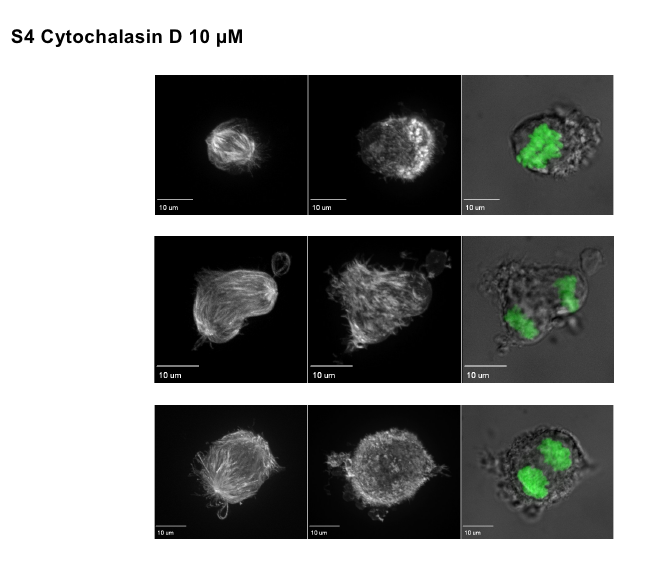

Supplement: Figure S4 — Cells treated with the actin polymerisation inhibitor cytochalasin D. Staining was for tubulin (left column), actin (middle column) and chromosomes (right column, overlay with DIC image). The pictures clearly show the expected disturbance of the actin cortex as well as the inability of the cells to undergo cytokinesis. (TIFF) [file pone.0028965.s006.tiff]

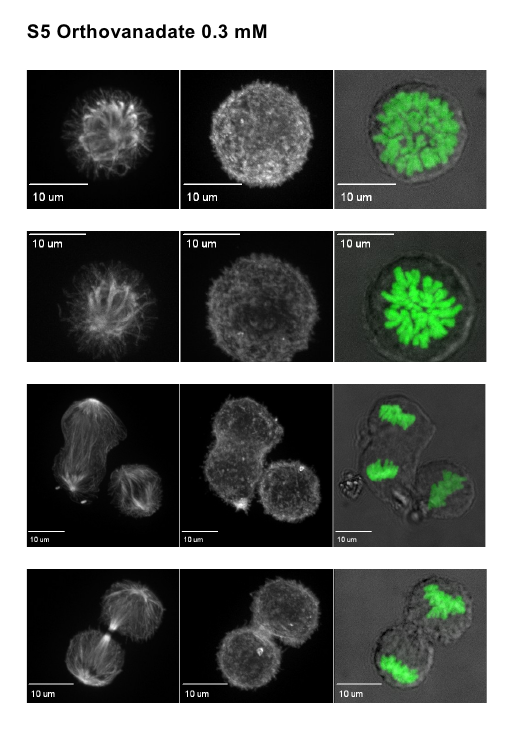

Supplement: Figure S5 — Cells treated with orthovanadate in prophase (top row), prometaphase (second row), metaphase & anaphase (third row) and telophase (bottom row) which had been treated with orthovanadate. Staining was for tubulin (left column), actin (middle column) and chromosomes (right column, overlay with DIC image). The different phases of mitosis appear normal. (TIFF) [file pone.0028965.s007.tiff]

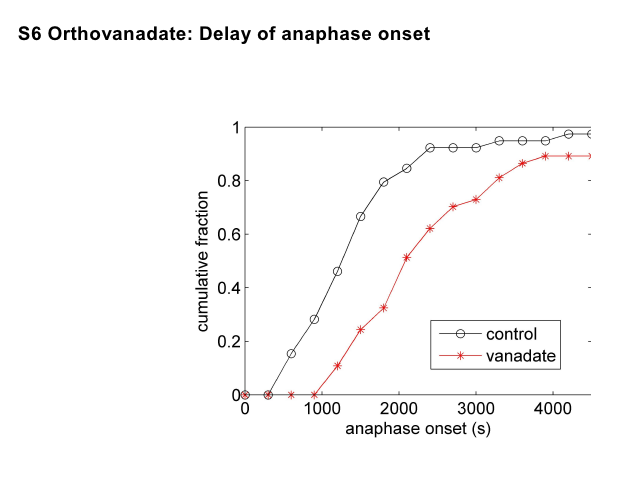

Supplement: Figure S6 — Orthovanadate treatment leads to a significant delay in anaphase onset. (TIFF) [file pone.0028965.s008.tiff]

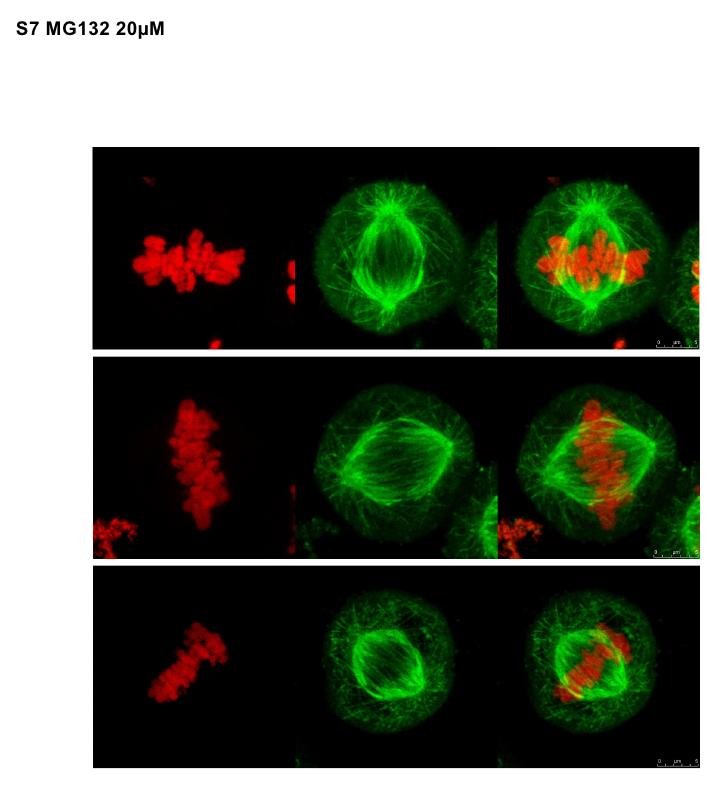

Supplement: Figure S7 — Uncompressed RPE1 cells stained for DNA using YO-PRO-1 (Invitrogen, Darmstadt, Germany) (left column) and tubulin (middle column). Right column: overlay, scale bar 5 µm. MG132 (Z-Leu-Leu, Sigma-Aldrich) is a proteasome inhibitor and prevents the mitotic cell from entering anaphase without affecting the mitotic spindle. These images are projections of a 3 µm z-stack around the cells centre obtained with a confocal microscope. (TIFF) [file pone.0028965.s009.tiff]
